# Adapted clustering method for generic analysis of histological fibrosis staining as an open source tool

**DOI:** 10.1038/s41598-023-30196-9

**Published:** 2023-03-16

**Authors:** Anca Remes, Marie Noormalal, Nesrin Schmiedel, Norbert Frey, Derk Frank, Oliver J. Müller, Markus Graf

**Affiliations:** 1 Department of Internal Medicine III, University Hospital Schleswig-Holstein, Kiel and German Centre for Cardiovascular Research, Partner Site Hamburg/Kiel/Lübeck, Kiel, Germany; 2 Department of Internal Medicine III, University Hospital Heidelberg, Heidelberg, Germany; 3 Faculty Industrial and Process Engineering, Heilbronn University of Applied Sciences, Heilbronn, Max-Planck-Str. 39, 74081 Heilbronn, Germany

**Keywords:** Cardiac hypertrophy, Software

## Abstract

Pathological remodeling of the extracellular matrix is a hallmark of cardiovascular disease. Abnormal fibrosis causes cardiac dysfunction by reducing ejection fraction and impairing electrical conductance, leading to arrhythmias. Hence, accurate quantification of fibrosis deposition in histological sections is of extreme importance for preclinical and clinical studies. Current automatic tools do not perform well under variant conditions. Moreover, users do not have the option to evaluate data from staining methods of their choice according to their purpose. To overcome these challenges, we underline a novel machine learning-based tool (FibroSoft) and we show its feasibility in a model of cardiac hypertrophy and heart failure in mice. Our results demonstrate that FibroSoft can identify fibrosis in diseased myocardium and the obtained results are user-independent. In addition, the results acquired using our software strongly correlate to those obtained by Western blot analysis of collagen 1 expression. Additionally, we could show that this method can be used for Masson’s Trichrome and Picosirius Red stained histological images. The evaluation of our method also indicates that it can be used for any particular histology segmentation and quantification. In conclusion, our approach provides a powerful example of the feasibility of machine learning strategies to enable automatic analysis of histological images.

## Introduction

Despite recent progress in diagnostics and treatment options, cardiovascular disease remains a leading cause of death worldwide^1^. One of the hallmarks of cardiovascular complications is the activation of pro-fibrotic processes^2^. Extracellular matrix deposition is associated with ischemic heart disease^3^, diabetic cardiomyopathy^4^ and hypertension^5^, accounting for reduced ejection fraction due to tissue stiffening and abnormal electric conduction leading to arrhythmias^6^. Although development of fibrosis is initially a protective mechanism of wound healing and regeneration, increased fibroblast proliferation and collagen deposition in the chronic stage of the disease were proven to lead to impaired cardiac diastolic function^6^. Preclinical studies revealed various mediators and possible therapeutic targets to be involved in promoting myocardial fibrosis^7,8^. Interestingly, the degree of fibrosis generation was proven to be a prognostic of cardiovascular events in patients^9^. These observations underline the need to accurately and selectively quantify extracellular matrix deposition in animal models and patient biopsies in the context of cardiovascular disease.

One of the most widely used methods to determine the degree of extracellular matrix deposition is staining of frozen or paraffin-embedded sections with specific dyes, such as Masson’s Trichrome or Picosirius Red. Although several methods have been employed to semi automatically quantify acquired images, proper validation of these approaches in animal models of cardiac dysfunction has not yet been performed^10–13^.

Hence, here we describe a novel staining quantification tool (FibroSoft) that was developed specifically to fit the aforementioned needs. Hereby, it is intended to decrease the time and effort of the challenging quantification process, especially by separating background, healthy, and fibrotic tissue areas. For that purpose, our proposed software is applying a modified supervised k-means clustering algorithm^14^. We therefore demonstrate the composition, accuracy and performance of this approach and show its relevance in an animal model of cardiac hypertrophy and heart failure.

## Methods

### Animal experiments

Animal experiments were approved by the local animal welfare committee in Schleswig–Holstein (Ministerium für Energiewende, Klimaschutz, Umwelt und Natur, permission number V312-7224.121–4). Mice were housed in pathogen-free, temperature and humidity-controlled environment, with ad libitum access to water and food. All efforts were undertaken to minimize animal suffering.

Transverse aortic constriction (TAC) was performed in 8 weeks old male C57BL/N 6 mice (Charles Rivers Laboratories) as previously described^15^. Twelve mice were used for each group, and no mortality was observed during the observation time. In brief, mice received intraperitoneal injection of Buprenorphine (0.1 mg/kg) for pain relief and were anesthetized with 2.5% isoflurane in 0.2 L/min of oxygen by oral intubation with a 20-gauge (G) tube and ventilation (Harvard Apparatus) at 120 breaths per minute (0.2 mL tidal volume). First, a small incision was performed in the second intercostal space to expose the aortic arch. A ligation was next positioned with a Prolene 6–0 suture between the brachiocephalic and arteria subclavia. For the spacer, a 27-G needle (Ø 0.42 mm) was used. Finally, the chest was closed with a suture (Prolene 4–0). The sham operation was performed similarly, excluding the ligature of aortic arch.

Echocardiography was conducted 6 weeks after surgery (VisualSonics Vevo 1100 and MS400 cardiovascular probe,18–38 MHz). For assessment of cardiac function, left ventricular (LV) fractional shortening (FS) and ejection fraction (EF) were measured by B-mode long axis. Additionally, the following parameters were measured by M-mode short-axis on the level of papillary muscles: LV interventricular septal thickness (IVS), LV internal dimensions (LVID) and posterior wall (PW) thicknesses at diastole and systole (IVSd, LVIDd, PWd and IVSs, LVIDs, PWs). Mice were sacrificed by cervical dislocation and cardiac tissue was collected 6 weeks after surgery. A total number of 12 mice were used for analysis in each group. Mice were randomly assigned to each group.

### Histological staining and image acquisition

Fibrosis staining was performed in 7 µm-thick frozen or paraffin tissue sections. Picosirius Red staining was conducted according to standard protocols^16^. Masson’s Trichrome staining was performed according to the instructions provided by the manufacturer (Sigma-Aldrich). Specimens were imaged using a brightfield microscope using a 20 × objective (BZ-X800, Keyence).

### Western blot analysis

Tissue lysates were prepared according to standard protocols with slight modifications^15^. In brief, 1 mL of RIPA buffer containing 0.1% sodiumdodecylsuphate (SDS) with phosphatase and protease inhibitors (Roche Diagnostics) was added to approximately 50 mg of heart tissue. The tissue was further homogenized and protein concentration was determined using DC assay (Bio-Rad). Tissue lysates containing 30 μg protein/well were further subjected to SDS-PAGE separation and proteins were transferred to PVDF membranes. Next, membranes were blocked with 5% skimmed milk. Further, membranes were cut at 55 kDa and primary antibodies was applied separately on each section of the membrane (upper part: Collagen 1a1, Santa Cruz, sc-293182, dilution 1:500; lower part: β-actin, Thermo Fischer Scientific, PA1-183, dilution 1:10 000), and incubated at 4 °C for 16 h. After a series of washes in TBS-T, a corresponding HRP-labeled secondary antibody (Dianova, dilution 1:10 000) was next incubated for 1 h at room temperature. Chemoluminescence was detected by Pierce ECL Substrate and using a ChemiDoc Imaging system (BioRad). Ponceau staining (BioRad) was used to visualize protein transfer. Relative protein levels were determined by ImageJ (NIH, version 1.8.0_66).

### Immunohistochemistry

Immunohistochemistry was performed to assess the levels of fibronectin in cardiac tissue sections. Seven μm-thick frozen sections were subjected to fixation with a solution containing 4% paraformaldehyde for 10 min, followed by blocking with 10% goat serum (Thermo Fischer Scientific) containing 0.01% Triton X-100 for 1 h at room temperature. Next, primary antibody (anti-fibronectin, Abcam, ab2413 1:400 in blocking buffer) was incubated overnight, in a humidified atmosphere. After a series of washes in PBS, secondary antibody conjugated with Alexa-546 (Thermo Fischer Scientific, 1:400) was added and incubated for 1 h at room temperature. Imaging was achieved using confocal microscopy (LSM 800, Zeiss), and mean fluorescence intensity was measured using ImageJ (NIH, version 1.8.0_66). Twelve images were analysed/experimental condition.

### Image processing and analysis

Our suggested approach and software implementation relies on pixel-wise tissue classification followed by quantifying the surface of specific tissue in comparison to a reference area (usually a cell). Here, the reference (cell) is defined as the part of the slice having background area removed. Pixel classification is done by applying a variation of the k-means clustering algorithm^17^. Colors are represented in HSV color space (hue, value, saturation instead of red, green, and blue intensities) that models color more closely to human vision. We use the HSV color space model to appropriately interpret boundaries and textures within the algorithm.

### Software architecture and design

FibroSoft is written in C++ using QT framework and deployed as an open-source software system with binaries available on current MacOS, and Microsoft Windows (64 bit) operating systems. Images are loaded as TIFF from a folder into a list to be processed. The user interface allows users to load, prepare, and monitor the analysis flow, however, in this section we focus on the implementation of our suggested k-means clustering approach. Therefore, analysis by modified multi-subclass k-means as described above is shown in pseudo code in Listing 1. The C++ implementation can be found in the open-source repository at sourceforge. This part of the code described is listed in *staininganalysis.cpp*.

Calculation is therefore moved into this specific class implementation in order that either the batch process or a single image can be clustered by the same algorithm.

The other code mainly consists of preparation and back-ground processing tasks, in order to make a more responsive user feeling. Results of batch processing can be exported to a comma separated value (.csv) file in order to postprocess within spread sheet calculation software tools or import it into statistical analysis programs.

### Development of the quantification tool and experimental setup

**Figure 1:**
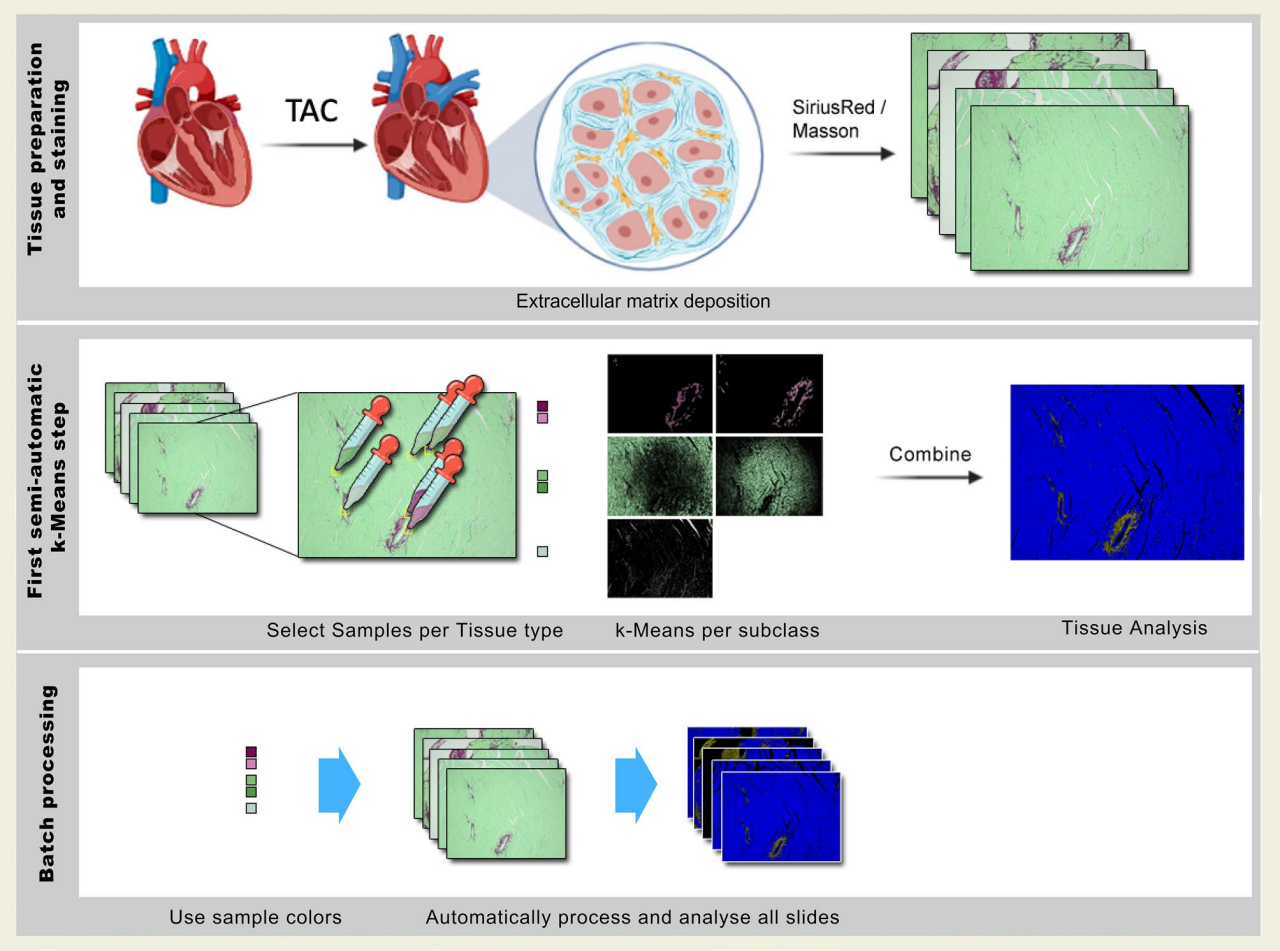
Description of the animal experiments, image analysis and software design.

**Figure 2:**
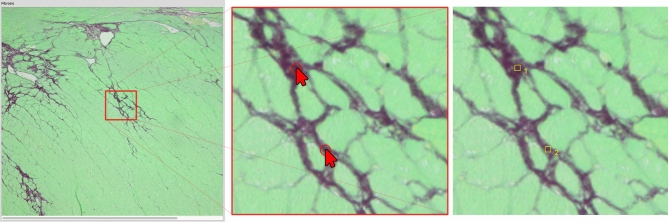
Quantification tool with sample selection panel and one sample for each tissue selected.

**Figure 3:**
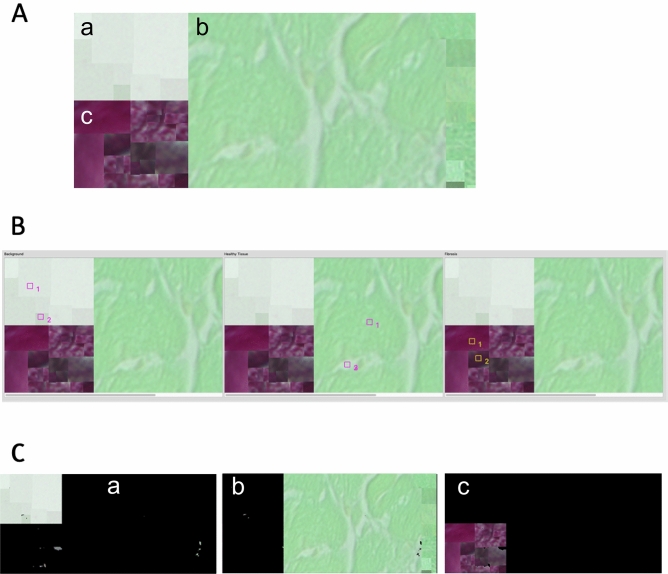
Schematic representation of the workflow and analysis process within the quantification tool.

FibroSoft ensures a semi-automatic quantification of any histological staining which means its semi-automatic processing isn’t bound to handle specific color spectrums. A summary of the animal experimental design and software workflow is described in Fig. 1. Moreover, Fig. 2 shows the initial sample selection step for the semi-automatic supervised clustering approach to evaluate fibrosis to cell ratio for quantification. FibroSoft’s main graphical user interface is split into three tissue preview panes where the user can interactively select sample points (color references) for the entire classification process. Figure 2 exemplarily shows the selection process for fibrotic tissue sample points. This is done for all tissue types as well as the background (in order to remove background pixels). After this step is done and the process is started, tissue clusters are computed automatically by cumulating corresponding *k*-means clusters for each class (tissue type sample). Another option is available to let the results be determined fully automatically on behalf of apriori knowledge coded into the application depending on the underlying staining method. After loading images to be processed, users have the option to either manually initialize the supervised k-means initial cluster centers by taking sample points for each tissue type, or to use pre-set data for each specific staining protocols (e.g., Masson’s Trichrome, Picosirius Red). Moreover, previously selected/estimated clusters can be subsequently used to create new segmentation results on other images of the same dataset for the aforementioned three tissue types. Figure 3 shows the workflow including interactions and decisions by users in order to quantify data sets. Once an appropriate setting is found, it can be used as a general pre-set fitting for a specific dataset within the same session and enables processing the entire experiment dataset in a single batch/run. Hence, the comparability between slices within the same experiments and specific data sets is additionally maximized.

This semi-automatic machine learning-based fibrosis classification approach is based on a slightly adapted *k*-means clustering algorithm which uses the features hue, saturation, and value of the HSV color space information. The adaptation can be described as a single-step supervised *k*-means with subclasses, as for each class users can choose multiple sample inputs. After classification corresponding subclasses are unified accordingly to the classes to be predicted. According to the initial definition of the k-means clustering method, all pixels are assigned to their closest cluster centers of the corresponding classes.

Instead of recalculating the cluster center µ_j within the cluster C_j after adding further sample points, we rather add new sub-clusters C_(j, i) to the list (described in Formula 2 and 3 below). All sub-cluster results will in the end be cumulatively added to the main segmentation S_j for that specific class/label l_j after the classification step.

According to Formula 4, the corresponding cluster sets for each tissue segmentations are combined (united) after they have been successfully identified in the *k*-means execution. With this approach we don’t average the sample point selections rather we add the range of each individually selected sample color. Therefore, the clusters will not be averaged and reassigned and we expect a more accurate adaption of multiple selections made by the user (Formula 4).

### Statistical analysis

Statistical analysis was performed using R^18^ and GraphPad Prism 5. Graphs are presented as mean ± SD. Statistical significance was calculated by Mann Whitney *U* test. *P* values smaller than 0.05 were considered as significant.

### Informed consent

All methods were performed in accordance with the relevant guidelines and regulations. The study is reported in accordance with ARRIVE guidelines. Images were created with BioRender.com.

## Results

### Single step supervised *k*-means with subclasses retrieves more reliable results than applying standard *k*-means

**Figure 4:**
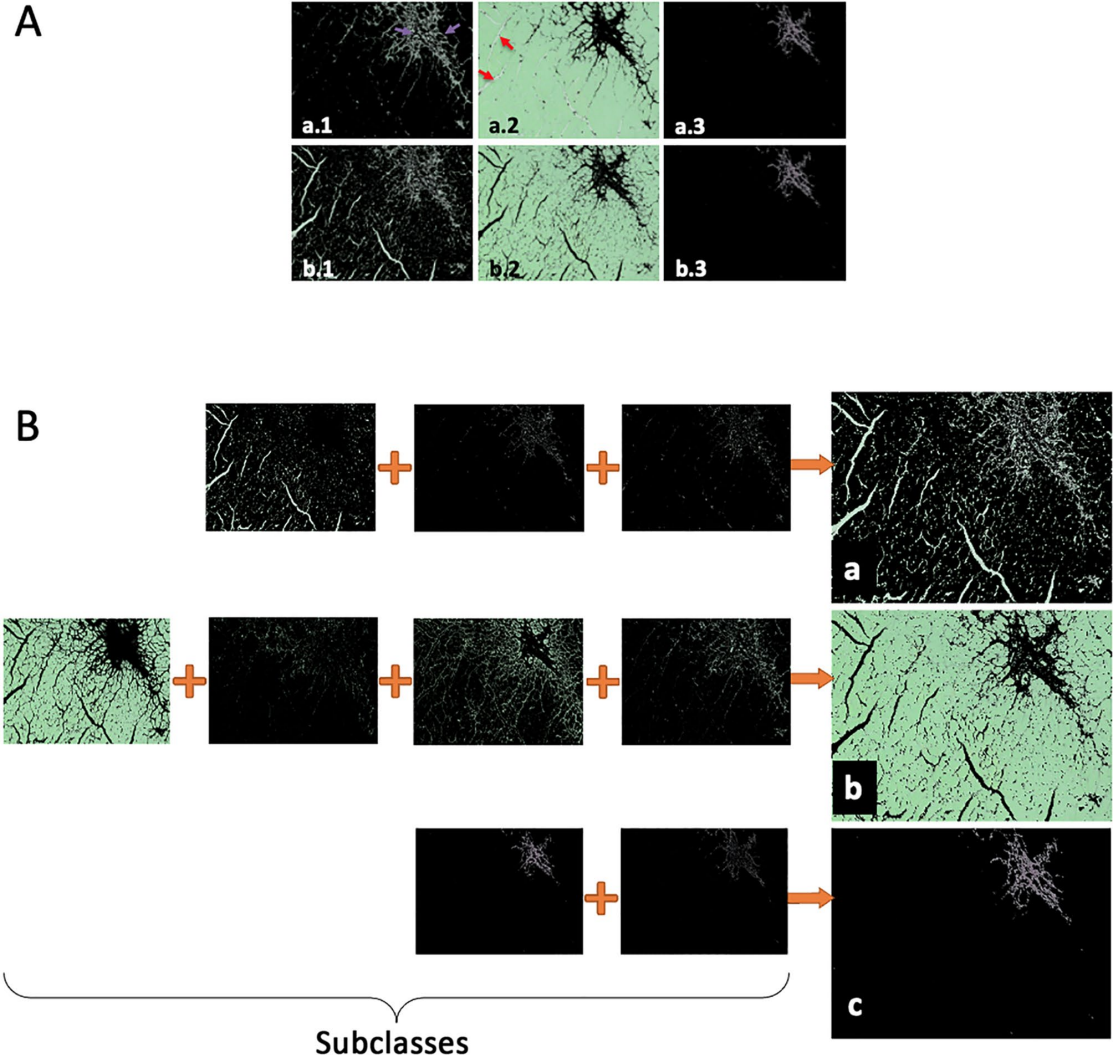
Workflow showing *k*-means classification with subclasses. (**A**) Standard k-means clustering results (a) compared to single step supervised *k***-**means (b) with each of the three classes (1–3). Arrows depict partially falsely classified tissue, in a.1 healthy tissue is shown as background (yellow arrows), where background was falsely classified as healthy tissue in a.2. (**B**) Supervised single step *k*-means with subclasses (= multiple samples for each tissue type) showing improved combined results on all tissue types (a) background pixels, (b) healthy tissue, and (c) fibrotic tissue.

Applying a standard *k*-means algorithm leads to repeatedly changing cluster centers around which the classified tissue will be arranged. Due to the fact that following histological processing some color ranges of different tissue types are hardly fully-automatically distinguishable, a slight shift within those cluster centres might lead to false results (as marked with arrows in Fig. 4A—algorithm a). A solution to this challenge is to use supervised cluster centers, whereby a sample point is given by the user as a reference. Moreover, restricting the algorithm to a single iteration of the k-means method will only cluster around those user-selected references.

As outlined in Fig. 4, b1 and b2, this method retrieves more accurate results than standard unsupervised *k*-means. However, there are many instances where too few pixels are identified as being healthy tissue and vice versa because of the usual iterative approach of this method. In order to solve this issue, we created a supervised modification that defines subclasses in such a way, that each tissue can be assigned to multiple subclasses which in the end are combined into the resulting class. Visual results and the complete working process are illustrated in Fig. 4B.

Due to the nature of *k*-means clustering, each pixel is assigned to only one result group. Therefore, Fig. 4B depicts the processing with multiple selections for each tissue assigned pixels in individual subsets which are then combined to retrieve the resulting final classifications a, b, and c.

### FibroSoft can effectively assess extracellular matrix deposition in TAC model of cardiac hypertrophy and heart failure

**Figure 5:**
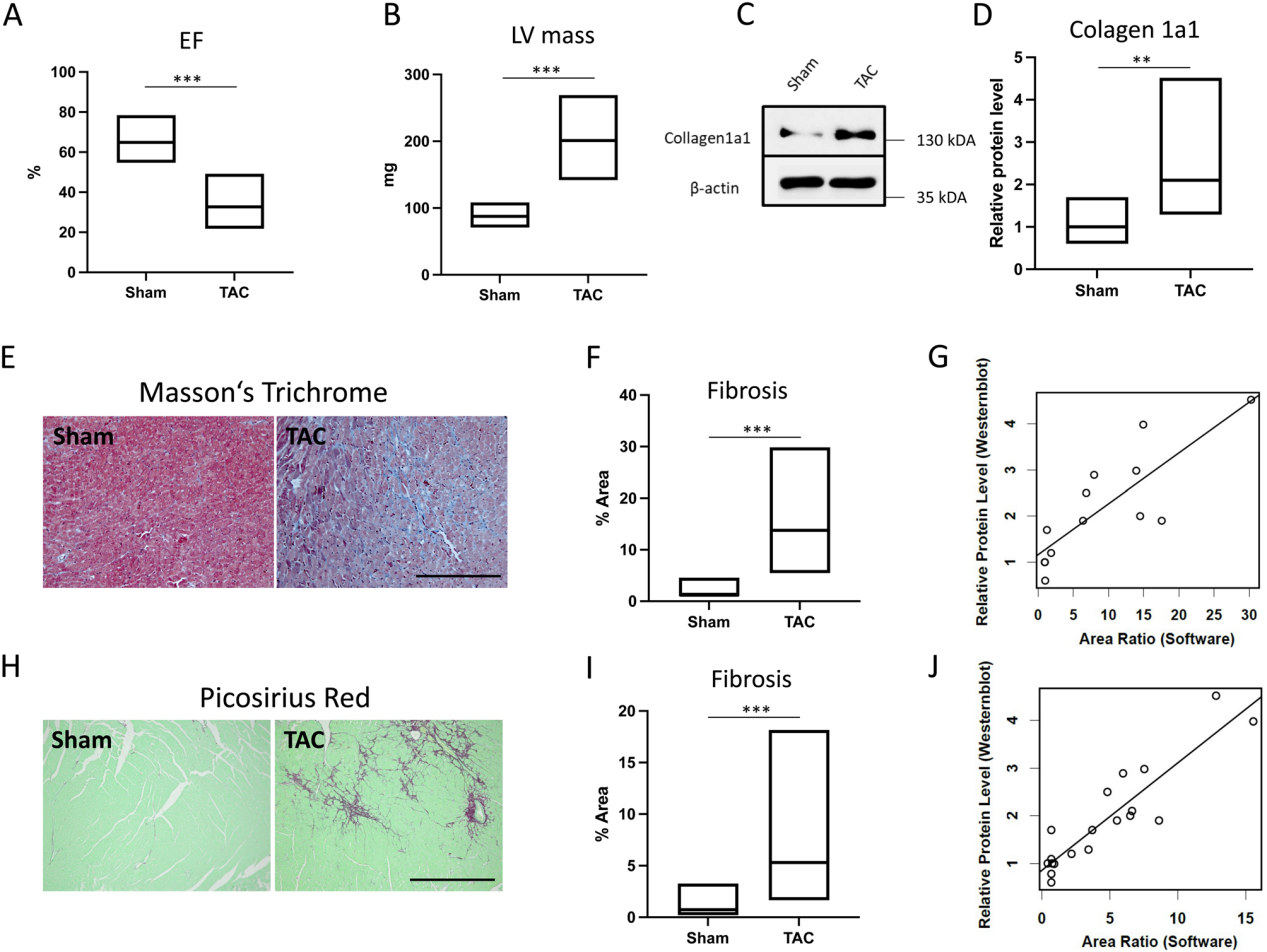
The results obtained by using the FibroSoft software strongly correlate with the results of Western blot analysis of collagen 1 in TAC model of heart failure. (**A**) Reduced cardiac function following TAC surgery in mice, measured by echocardiography. (**B**) Statistical quantification of left ventricular mass in mice subjected to TAC. (**C**, **D**) Analysis of collagen 1a1 expression in myocardium of mice by Western blot. Tubulin served as a loading control, proving equal loading of protein samples. Values were normalized to sham operated mice. (**E**) Representative images showing Masson’s Trichrome staining of cardiac paraffin sections in TAC and sham operated mice. Blue areas represent extracellular matrix deposition, while red stained tissue accounts for heathy heart muscle. (**F**) Statistical quantification of percentage fibrosis area in Masson’s Trichrome staining images, quantified by FibroSoft. (**G**) Scatter plot showing in each sample relative protein level against the surface percentage (**H**) Illustrative images showing Sirius red staining of cardiac sections in sham and TAC mice. (**I**) Quantification of percentage fibrosis area in histological sections subjected to Sirius red staining. (**J**) Scatter plot analyzing correlation between Western blot analysis and FibroSoft in sections subjected to Sirius red staining. EF: ejection fraction, LV: left ventricle. (n = 12 mice/group, 12 images analysed, ***p < 0.001, Mann–Whitney *U* test).

Next, we aimed to investigate whether our results obtained with the designed software correlate with Western blot analysis of collagen 1 expression in sham and TAC-subjected mice. As expected, TAC caused severe deterioration of heart function in mice, suggested by significantly decreased EF and increased LV mass, measured by echocardiography (Fig. 5A,B). In addition, we could detect a marked elevation of collagen 1 expression in myocardium following TAC as compared to respective controls (Fig. 5C,D).

Further, we analysed the fibrotic tissue segmentation for Picosirius Red and Masson’s Trichrome staining using FibroSoft and compared it to the values obtained through Western blot analysis. We particularly focused our analyses on the left ventricle, since pressure overload induced by TAC induces left ventricular remodeling and dysfunction. As depicted in Fig. 5E,F,G,H,I,J and Table 1, the use of our designed software proves a significant difference between fibrosis levels in sham versus TAC cardiac left ventricular samples of a similar magnitude as Western blot analysis. Moreover, fibronectin levels followed the same pattern, as demonstrated by immunohistochemistry experiments (Supplementary Figure. 4). To compare the feasibility and performance of our software we analyzed histology staining of each mouse accordingly to its corresponding Western blot data set. Most importantly, the values obtained with the two independent methods strongly correlate, evaluated in individual mice (Fig. 5F,I, Table 2).

**Table 1:** Statistical analysis of differences between sham and TAC tissue.

Analysis	n sham	n TAC	W	*P*
Western blot	11	8	86.5	$0.0005154$
Picosirius red	24	33	790	$1.064\cdot {10}^{-15}$
Masson’s trichrome	21	33	693	$8.161\cdot {10}^{-10}$

**Table 2:** Regression analysis of scatter plots presented in Fig. 5F,I.

	Picosirius red/Western blot	Masson’s trichrome/Western blot
R^2^	0.8276	0.6804
*p*	$6.727\cdot {10}^{-08}$	0.00052

Furthermore, as demonstrated in Supplementary Figure. 1, interindividual segmentation results and quantification were equally distributed between observers for both Masson’s Trichrome and Picosirius red staining. Therefore, we can conclude that FibroSoft shows robust behavior when managed by different users.

### Analysis of defined reference volume shows high accuracy of FibroSoft

In order to assess the accuracy of any newly created software one usually compares it against an existing gold standard. However, as there is no widely used method besides manually extracting tissue areas by applying various imaging techniques^19^ and as those manual analysis methods are very time-consuming and prone to subjectivity, we defined a reference slice combining randomly selected sets of samples from all our available microscopy images per tissue type as well as background. Then, we analyzed the performance of the software against this standard reference image expecting each tissue specific compartment (a, b, and c) to be coherently classified and masked by its type (i.e., execting rectangular shaped areas, Table 3).

**Table 3:** Volume comparison by tissue type.

Tissue type	Defined surface (px)^2^	Analyzed (FibroSoft) (px)^2^	Dice coefficient between defined and analyzed
Background	21,336	21,404	0.9948
Healthy	107,188	107,141	0.9972
Fibrotic	21,336	21,315	0.9876

**Figure 6:**
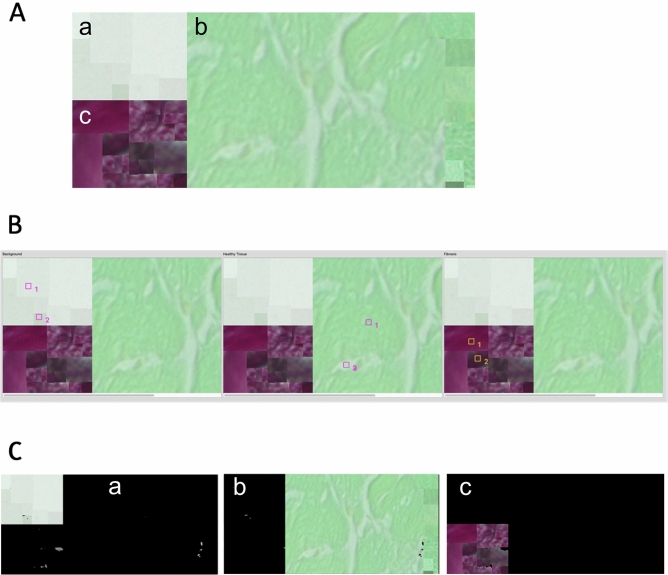
Reference chart and results of accuracy analysis. (**A**) Defined reference chart which combines randomly selected samples per tissue type and then arranged for accuracy evaluation: (a) healthy tissue, (b) fibrotic tissue samples, (c) background samples. (**B**) Selected sample color points for reference tissue associated subclasses for our *k*-means algorithm. (**C**) Resulting image masks of FibroSoft cluster analysis using supervised *k*-means with subclasses obtained from a run on the defined reference image; (a) identified as healthy tissue, (b) classified fibrotic tissue, and (c) background.

For that purpose, this rectangular test image, displayed in Fig. 6A was analyzed using FibroSoft tool after specifying individual tissue associated subclasses as shown in Fig. 6B by the indexed squares. Results of this analysis show a ratio of 16.54% between fibrosis and the entire cell (as fibrosis + healthy tissue). The defined ratio of our reference setup is expected to be 16.56% (21,336 px^2^/128,835 px^2^).

Moreover, Table 3 proves an accuracy of around 99% measured by dice coefficient in all tissue types. Figure 6C depicts a clear and correct separation of the specified tissues by applying our supervised *k*-means with subclasses algorithm on the test image.

## Discussion

Here we underline a novel tool for semiautomatic analysis of cardiac fibrosis that allows quantification of both Picosirius Red and Masson’s Trichrome stained tissue, validated in a widely used murine model of cardiac hypertrophy transitioning to heart failure. In addition, we prove that FibroSoft is efficient not only in determining extracellular matrix deposition in heart tissue, but also in lung paraffin and frozen sections, and could further be applied on various tissue types and disease models.

Western blot analysis can accurately determine the pathological degree of collagen deposition; however it does not allow assessment of the location of extracellular matrix in pathological conditions. This is particularly important for cardiovascular diseases, in order to distinguish the types of fibrosis, namely perivascular, interstitial or replacement^2^, all leading to impaired heart function and high risk of arrhythmias. Therefore, it is critical that users can precisely quantify and analyse the areas of interest within the tissue section.

An utmost important result of our study is the strong correlation of collagen 1 content, quantified by Western blot, with the results of the designed software. Collagen 1 was demonstrated to be highly increased during the transition between compensated cardiac hypertrophy to heart failure, as well as in biopsies isolated from patients diagnosed with dilated cardiomyopathy^20^. Moreover, collagen 1 confers tissue rigidity, leading to impaired cardiac function in several models of cardiac dysfunction^21^. Although collagen is the most abundant extracellular matrix protein in the heart, abnormal deposition of fibronectin was proven to contribute to pressure overload-induced myocardial dysfunction^22^. Interestingly, inhibiting fibronectin polymerization was shown to be beneficial in a mouse model of cardiac dysfunction and might be translated into a therapeutic option for heart failure^23^. Our data demonstrate that fibronectin levels also correlate with the data analysed by FibroSoft, suggesting that not only collagen can be accurately measured through this method, but other molecular players with major role in fibrosis development in heart failure.

Another benefit of our software is that it enables the quantification of both Picosirius Red and Masson’s Trichrome-based histological stainings, allowing users to choose according to the tissue or disease of interest. For example, Picosirius red was proven to be more accurate in quantification of hepatic fibrosis in patients with hepatitis C^24^. Similarly, renal fibrosis, quantified by Picosirius red staining correlates stronger with kidney function than Masson’s Trichrome and collagen staining in renal biopsies^25^.

Furthermore, FibroSoft allows users to manually choose extracellular matrix color hue, which was shown to differ with the progression of disease and age due to changes in the 3D structure of collagen protein^26,27^.

In conclusion, we prove the feasibility of machine learning methods (here: supervised k-means with subclasses) to design a semi-automatic fibrosis software, focusing on Masson’s Trichrome and Picosirius Red staining methods, which improves the specificity and reduces human error compared to previously described methods in the field. Additionally, the resulting final classifications contain smoother differences due to multiple cluster centers per class.

Unlike other procedures, FibroSoft discriminates between background tissue without relying on any kind of specific threshold values or preprocessing steps. Thus, this software is suitable for the analysis of Masson’s Trichrome and Picosirius Red staining as well as other histological analyses whenever three classes need to be distinguished.

Moreover, we can demonstrate that FibroSoft can clearly distinguish between healthy and diseased myocardium in case of pressure overload-induced cardiac hypertrophy and heart failure.

To the best of our knowledge, other available tools don’t provide similar functionality or lack the proof of accurate performance. Furthermore, several Photoshop-based analysis methods do neither prove the accuracy of the segmented results, nor are suitable for other staining techniques^13^.

Therefore, this tool may become useful not only for basic research-based studies, but could be translated into a powerful tool for tissue biopsy analysis not only in cardiovascular complications, but other disorders affected by abnormal extracellular matrix deposition. Its simple user interface and straightforward parametrization approach as well as batch processing create an ease of use for image analysis in various settings, for example under individual staining, disease models, color-shifts and lighting conditions. Thus, it can be widely and easily applied to yet even unknown analysis tasks.

Moreover, first experiments with deep learning algorithms fed by data produced with FibroSoft have shown that a fully automatic classification system is at reach in order to remove complete interaction. Further research is required to translate this tool into a fully automatic fibrosis classification software.

## Conclusion

In conclusion, here we underline a novel approach to automatically determine the degree of extracellular matrix deposition in cardiac sections under pathological conditions. Further studies assessing the efficacy of the method in human biopsies are required to translate the approach to patient situation.

## Supplementary Information

Supplementary Information.

## Data Availability

The datasets used and/or analysed during the current study available from the corresponding author on reasonable request.

## Abbreviations

CSV: Comma-separated values
EF: Ejection fraction
FS: Fractional shortening
H: Hour
HRP: Horseradish peroxidase
HSV: Hue, saturation, value
IVS: Interventricular septal thickness
L: Liter
LV: Left ventricle
LVID: Left ventricular internal dimensions
MHz: Megaherz
mg: Milligram
mL: Milliliter
mm: Millimeter
μm: Micrometer
μg: Microgram
PAGE: Polyacrylamide gel electrophoresis
PW: Posterior wall
SD: Standard deviation
SDS: Sodium dodecyl sulfate
TAC: Transverse aortic constriction
TBS-T: Tris-buffered saline-Tween
TIFF: Tagged image file format
°C: Degree Celsius

## Code availability

The software is available via sourceforge: https://sourceforge.net/projects/fibrosoft/.
