# Supplementary material for: Adapted clustering method for generic analysis of histological fibrosis staining as an open source tool
Source: Sci Rep. 2023 Mar 16;13:4389. doi: 10.1038/s41598-023-30196-9 (PMC10020481; doi:10.1038/s41598-023-30196-9)

Supplementary Fig. 1. Box plots proving that analysis with FibroSoft is quantifying tissue classification correctly in a user-independent as well as staining-independent way (n=12 mice/group).

Picosirius red

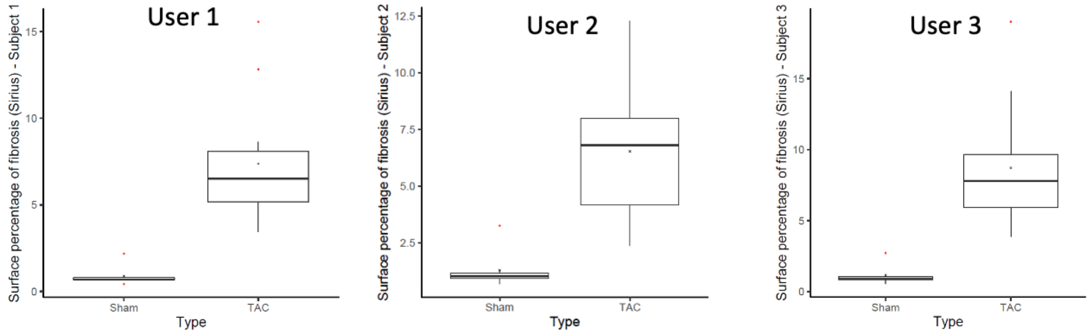

Masson’s trichrome

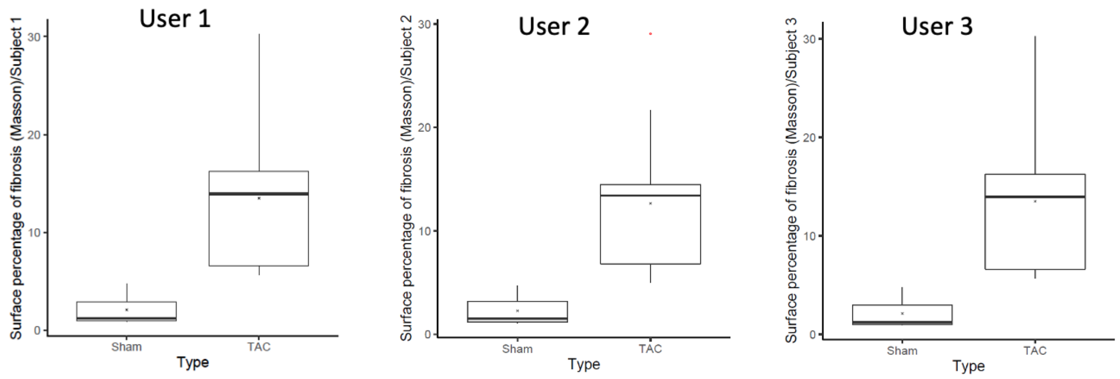

Supplementary Fig. 2. Quantification and classification steps from original image, image with background removed, classified healthy tissue and classified fibrotic tissue for two different cryosections in order to show its performance visually.

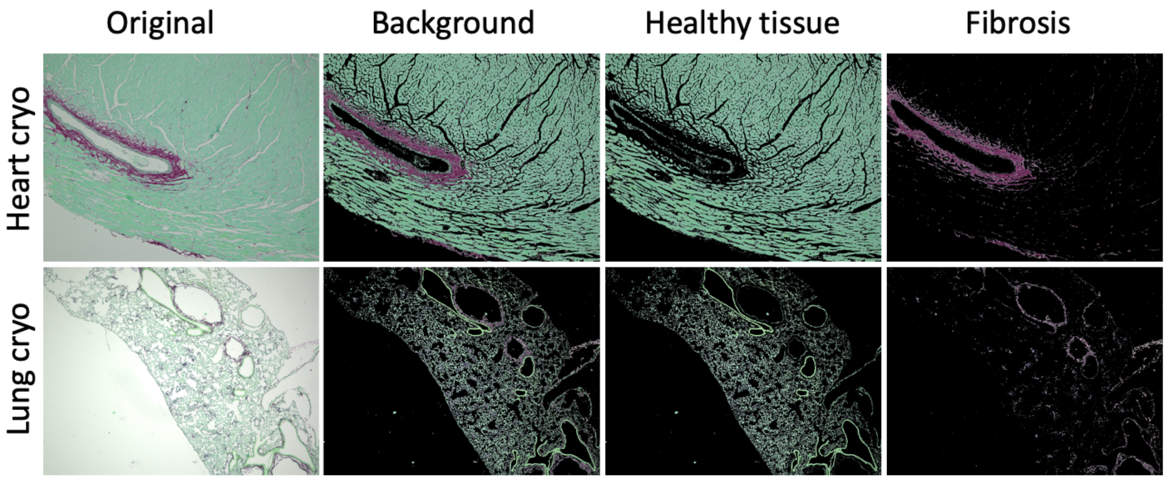

Supplementary Fig. 3. Raw images of Western blot analyses presented in the manuscript.

1. 2. 3. 4. 5. 6.

Collagen1A1

β-actin

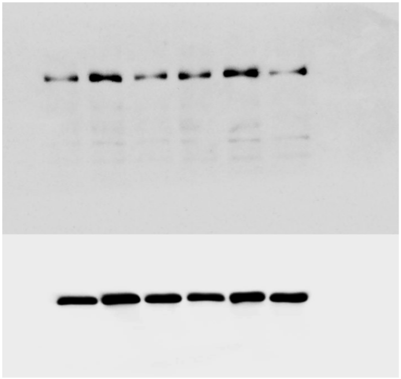

Mouse numbers (randomized):
1: Sham
2: TAC
3. Sham
4. Sham
5. TAC
6. Sham

Exposure time:
Collagen1A1: 30s
β-actin: 5s

Other exposure times (original, non-inverted frames):

Collagen1A1

β-actin

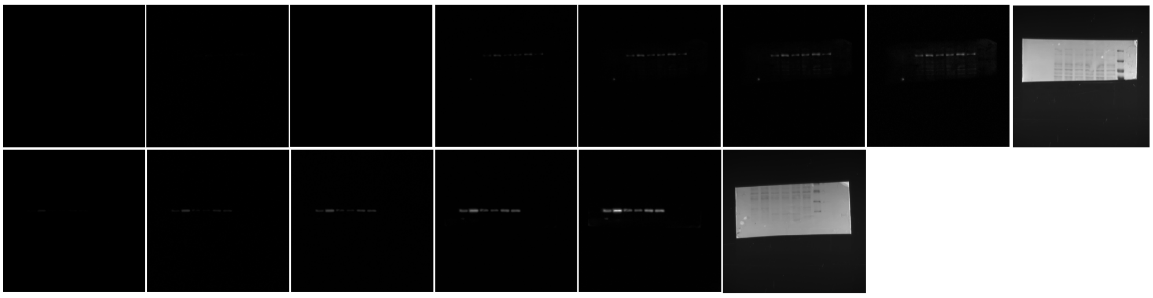

Supplementary Fig. 4. Quantification of fibronectin levels in TAC-treated animals. **(A)** Illustrative images showing fibronectin staining (red) in cardiac sections of mice subjected to the depicted treatment groups. DAPI (blue) was used as a nuclear marker. Scale bar represents 25 µm. **(B)** Statistical quantification of red fluorescence intensity in the sham and TAC sections, indicating relative fibronectin protein level.

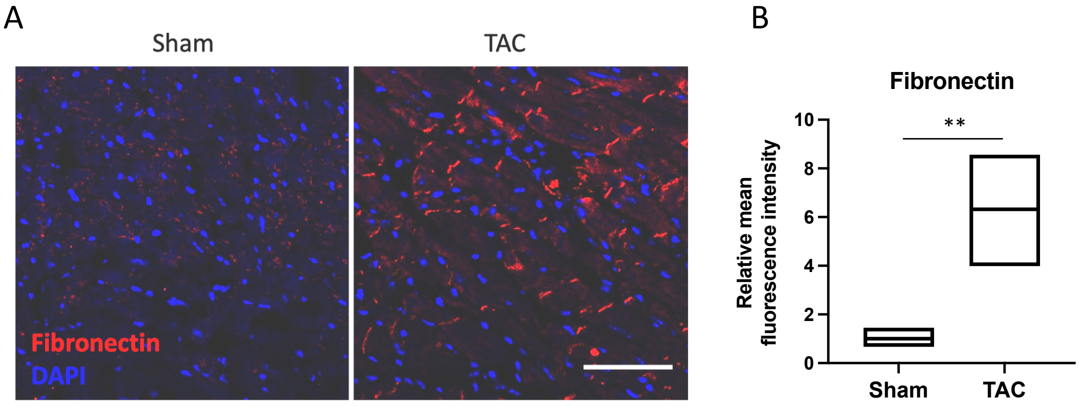

Supplement: Supplementary file 1 — Supplementary Information. [file 41598_2023_30196_MOESM1_ESM.pdf]
